# Harvard University, Veterinary Department

**Published:** 1896-09

**Authors:** 


### VETERINARY DEPARTMENT OF HARVARD UNIVERSITY.

OWING to the ill-health of Prof. C. P. Lyman, Prof. F. H. Osgood has been acting Dean during the past year. Harold C. Ernst, M.D., becomes full professor of bacteriology for the new year. Theobald Smith, Ph.D., M.D., will fill the chair of applied zoölogy, a subject of much importance to advanced veterinary education. Frederick A. Davis, M.D., instructor in physiology, retires, also A. H. Wentworth, M.D., assistant in chemistry. Langdon Frothingham, M.D.V., becomes assistant to the chair of pathology. Albert James Sheldon, D.V.S., instructor in meat-inspection, succeeds Alexander Burr, M.D.V., who retires.

Changes in the course of studies have been planned, and form and action, hygiene, histology, medical chemistry, and bacteriology have been added to the first-year studies. These changes are all in the line of keeping in advance of the needs of higher veterinary education, and exhibit a deep and confident interest in the great future of veterinary science in the wide field of sanitary medicine.

